# *Lactobacillus fermentum* and *Lactobacillus crispatus* Do Not Have Cytotoxic Effects on HN5 Oral Squamous Cell Carcinoma Cell Line

**DOI:** 10.1155/2021/3034068

**Published:** 2021-09-28

**Authors:** Sepideh Mokhtari, Saede Atarbashi-Moghadam, Elahe Motevaseli, Soudeh Ghafouri-Fard, Ardeshir Hesampour

**Affiliations:** ^1^School of Dentistry, Tehran University of Medical Sciences, Tehran, Iran; ^2^Dental Research Center, Dentistry Research Institute, Tehran University of Medical Sciences, Tehran, Iran; ^3^Department of Oral and Maxillofacial Pathology, School of Dentistry, Shahid Beheshti University of Medical Sciences, Tehran, Iran; ^4^Department of Molecular Medicine, School of Advanced Technologies in Medicine, Tehran University of Medical Sciences, Tehran, Iran; ^5^Department of Medical Genetics, Shahid Beheshti University of Medical Sciences, Tehran, Iran; ^6^Department of Biology, Central Tehran Branch, Islamic Azad University, Tehran, Iran

## Abstract

**Background:**

The oral environment has a very complex normal flora and a wide variety of bacteria including *lactobacilli*. Studies have shown oral microbial flora has important influence in the development of oral cancer. Squamous cell carcinomas account for more than 90% of cancers in oral cavity. *Lactobacilli* are known as one of the newest methods for the prevention and treatment of cancers. Previous studies on the effects of probiotics on oral cancer cells are very limited, and only two species of *Lactobacillus* which are not present in the normal oral microflora have been studied. Due to the unknown effects of *lactobacilli* on oral cancer, this study aimed to investigate the effect of two species of *lactobacilli* of oral cavity on oral cancer cells.

**Methods and Materials:**

The effects of the supernatant of two *lactobacilli*, namely, *fermentum* and *crispatus* were studied on HN5-cancer cells. The MTT method was used to study the effects of *lactobacilli* on inhibition of cancer cell growth.

**Results:**

The results showed that these *lactobacilli* do not prevent the progression of oral cancer cells. Moreover, the results showed that the acidic medium had the most effect on reducing the growth of oral cancer cells.

**Conclusion:**

Due to the different effects of *lactobacilli* on various cancer types, the effects of two *Lactobacillus crispatus* and *Lactobacillus fermentum* on other oral cancer cell lines may be different from what has been reported in this study.

## 1. Introduction

Squamous cell carcinomas (SCC) account for more than 90% of oral malignancies [1]. There are numerous risk factors for oral SCC such as alcohol and tobacco use, geographic variation, genetic predisposition, diets, immune status, oncogenic viruses, radiation, poor oral hygiene, and environmental factors [1, 2]. Survival of SCC patients over the past two decades has been only increased from 40% in 1950 to 59%. Therefore, this cancer has a poor prognosis and also a low survival rate. Despite many scientific advances about the cancer molecular mechanism, no significant progress has been made in the treatment and survival rate of SCC patients [1, 3]. Furthermore, control of oral SCC is difficult even after treatment because it has a tendency towards multiple primary carcinomas [4]. Its current treatment approaches include conventional therapies for cancer that have many side effects despite not having significant results. Therefore, modern pharmacology seeks treatments with low side effects and high efficacy. *Lactobacilli* are a type of bacteria that are found in most of the body mucosal tissues including the mouth. These bacteria have several strains with different distributions in various communities based on different conditions. They affect the expression of genes including oncogenes and tumor suppressor genes. In recent years, their preventive role in cancers has been noticed significantly. Studies have shown that *lactobacilli* can inhibit the function of intestinal cancers. Numerous studies have proven their role in preventing colon cancer [5, 6]. Therefore, in this study, we investigated the effects of two famous species of *Lactobacillus* called *L. fermentum* and *L. crispatus*.

## 2. Materials and Methods

### 2.1. First Stage: Culturing Oral Squamous Carcinoma Cells

HN5 squamous cell carcinoma line was purchased from Pasteur Institute. This cancer cell line has been obtained from the tongue squamous cell carcinoma of a 73-year-old man. Cells were cultured in the DMEM medium containing 15% glucose, 10% heat inactivated fetal calf serum (Invitrogen), 1.5% HEPES (Invitrogen), and 1% penicillin/streptomycin (Invitrogen). Cells were kept as monolayer cultures at 37°C in a humidified 5% CO_2_ incubator and were plated 24 h before treatment to permit their adherence.

### 2.2. Second Stage: Culturing *Lactobacilli*

*L. fermentum* strain 4–17 and *L. crispatus* strain SJ-3C-US were cultured in de Man Rogosa Sharpe (MRS) broth (Merck; pH 6.5) at 37°C for 24 h under microaerophilic conditions. Bacteria were cultured overnight. When appropriate cell deposition was observed and the number of bacteria reached around 10^9^ CFU/mL, they were placed in a refrigerated 5000 rpm centrifuge for 15 minutes at 4°C. Supernatants were filtered through a 0.2 mm membrane filter to eliminate the residual bacteria and their remnants. The resulting supernatants pH was measured and was then filtered and kept in the refrigerator. To appraise the effect of lactate synthesized by *L. fermentum* and *L. crispatus* and pH alterations on cells, the lactate levels of supernatants were checked using a Lactate Randox kit (Randox Laboratories).

### 2.3. Third Stage: MTT Assay

HN5 cells were obtained from the Pasteur Institute's cell bank and were cultured with the conventional method. HN5 cells were first centrifuged and counted; then, 100 *μ*L of DMEM culture medium containing 15% FBS and with a cell density of 10,000 cells was added to 96-well flat-bottom microplates. In other words, *lactobacilli* supernatants were added to cancer cells in different concentrations. The microplate was incubated for 48 hours; then, 200 *μ*L of 5 mg/10 mL MTT solution was added to each well. The plates were then incubated for another 4 hours. Finally, the formazan dye crystals deposited in the cytoplasm of the cells were dissolved by adding 100 *μ*L of DMSO to each well, and the color intensity was recorded by ELISA at 570 nm. MRS and MRSPH were used as controls. MRS was a *lactobacilli*-free culture medium. MRSPH was also a *lactobacilli*-free culture medium which was acidified with lactic acid to measure the effect of lactate accumulated in the environment as *lactobacilli* grow.

Cell viability was measured using the following equation:

(1) $$\begin{array}{c} \text{Viability}\left(\text{percentage of control}\right)=\left[\frac{\left(\text{absorbance sample}-\text{absorbance blank}\right)}{\left(\text{absorbance control}-\text{absorbance blank}\right)}\right]\times 100. \end{array}$$

### 2.4. Fourth Stage: Statistical Analysis

Analyses were performed in SPSS v.15.0.1 (SPSS Inc., Chicago, IL). The Mann–Whitney test was used to calculate the IC50 of cells exposed to *lactobacilli*. *P* < 0.05 was considered statistically significant.

## 3. Results

### 3.1. Effect of *L. fermentum* and *L. crispatus* Supernatants on HN5 Cell Growth

**Figure 1:**
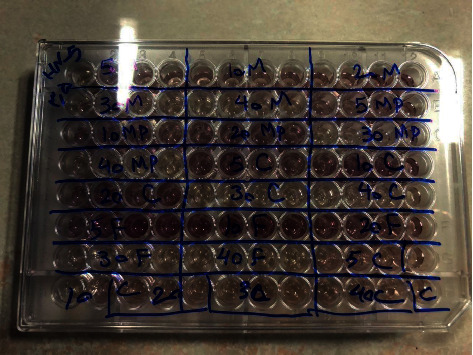
The results of the MTT assay for different concentrations of *L. crispatus* (C), *L. fermentum* (F), MRS (M), and MRSPH (Mp) on HN5 cells.

**Figure 2:**
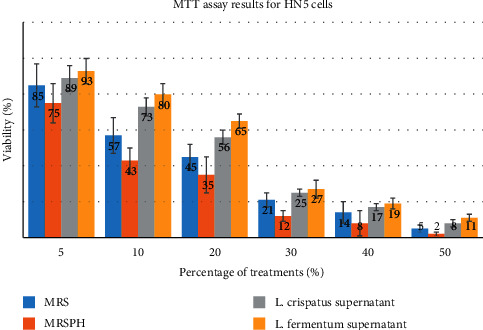
MTT assay results for HN5 cells.

**Figure 3:**
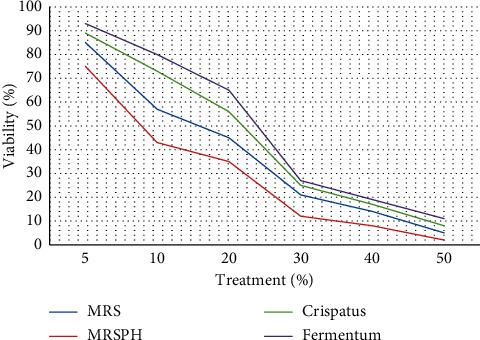
Linear curve of the growth inhibition rate of cancer cells in 4 different environments.

In this study, the inhibitory effect of *L. fermentum* and *L. crispatus* on HN5 oral cancer cells was evaluated by the MTT method.  Figure 1 shows the results of the MTT assay. IC50 was calculated for each environment that the cells were exposed to (Table 1). IC50 is a concentration of the medium that kills 50% of cells. As given in Table 1, IC50 is the lowest for MRSPH. In other words, the highest growth inhibition was observed in the MRSPH environment (Figure 2). As we know, MRSPH is a culture medium that has been acidified. So, the acid culture medium had the most lethal effect on cancer cells. According to the obtained values (Figure 3), the inhibitory effect of MRS and MRSPH (control media) culture media on the growth of cancer cells was more than the studied *lactobacilli*. In other words, although with the increase of *lactobacilli* concentration, the growth inhibition of cancer cells has been increased, and this growth inhibition has been more in control environments. Therefore, increasing the concentration of bacteria has not been the main factor in inhibiting the growth of cancer cells. So, *L. fermentum* and *L. crispatus* did not inhibit the progression of HN5 oral cancer cells.

## 4. Discussion

Cancer is the abnormal growth of cells. Uncontrolled cell proliferation and resistance to programmed death are major characteristics of cancerous cells. Therefore, factors that cause death (apoptosis or necrosis) in cancer cells can be regarded as anticancer agents [7, 8]. The incidence of SCC is associated with various factors, and this malignancy is a multifactorial disease in which both environmental and genetic factors play roles [1]. The oral environment is a special environment due to its constant contact with the environment outside the body, the presence of teeth, and its complex normal flora. There are more than 700 species of bacteria present in this environment. More than half of them have not yet been cultured. Some of these bacteria are involved in oral diseases such as caries and periodontal diseases. The presence of specific species of oral bacteria in systemic diseases such as bacterial endocarditis, pneumonia, pediatric osteomyelitis, low birth weight infants, and cardiovascular diseases has also been proven. However, researchers have little information about the microbial flora of the oral cavity. The oral microbial composition may be affected by a variety of factors, including oral health, genetics, age, sex, stress, and diet [9, 10]. Oral cancer cells are in constant contact with the microbial flora of the mouth. Therefore, cancer cells and the microbial flora of the mouth can interact with each other. Bjarnsholt et al. concluded in their research that poor oral hygiene leads to the formation of bacterial biofilm, and this biofilm contributes in oral cancers [11].

In a study by Hsiao et al., the association between increased growth of some oral bacteria caused by poor oral hygiene and the development of oral cancer was strongly established [12]. Wu et al. found that poor oral hygiene was one of the factors contributing to the poor prognosis of patients with oral cancer [13]. It is clear in the literature that the microbial flora of the oral cavity in patients with oral cancer is completely different from healthy individuals [14]. So, studies such as the present study are very effective in determining the exact relationship. The oral cavity microbial flora includes various types of bacteria such as staphylococci, streptococci, rods, and filaments. Different types of *lactobacilli* are also present in the mouth. *Lactobacilli* appear in the oral environment from the earliest years of childhood and are found in large numbers in saliva, mucous membranes, dorsal surface of the tongue, hard palate, and dental plaque, and even on dental surfaces in smaller numbers [15]. Extensive research has been performed on the relationship between *lactobacilli* and caries. The most important characteristic of *lactobacilli* is their ability to produce acid and their ability to grow and survive in an acidic environment. The latest results identify *lactobacilli* as secondary invaders rather than caries initiators. There are many different types of *lactobacilli* in dental caries. The presence of *L. fermentum* and *L. crispatus* has also been proven in adult dental caries [16].

In addition to the oral cavity, *lactobacilli* are present in other parts of the human digestive system and have beneficial effects for the host. Consumption of *lactobacilli* (as probiotics) leads to the production of a wide range of fermentation products such as high concentrations of short-chain fatty acids [17]. Studies show that *lactobacilli* have the anticancer effect, and they induce this effect through various mechanisms such as inducing an immune response and antiproliferative properties. It seems that one of the reasons for the toxic effects of *lactobacilli* on cancer cells is the high level activity of mitochondria in cancer cell respiration processes compared to normal cells, which provides an appropriate condition for the exploitation of these microorganisms to destroy cancer cells. Besides, it can be said that morphological differences and also the number of cell pores in cancer and normal cells are other factors to justify the increase in their cytotoxicity in cancer cells [18].

Investigations in oral SCC have also shown that microbiome can be influenced by radiotherapy, chemotherapy, and immunotherapy. Moreover, these treatments are influenced by the microbiome content [19]. Various laboratory studies have been performed on the use of *lactobacilli* in the treatment of various cancers, especially breast cancer, colorectal cancer, and cervical cancer [20]. However, studies on the effects of *lactobacilli* on oral squamous cell carcinoma have been very few and limited to two specific strains of *lactobacilli*, *Acetobacter syzygii* and *sp. A-2*. *Acetobacter syzygii* secretions have shown a significant cell-killing effect on oral mucosal cancer cells, and this effect has been much less on normal cells [21]. *Lactobacillus SP.A-2* also increased apoptosis and cell death in tongue cancer cells [22]. Certainly, applying the results of these research studies requires more detailed studies on the molecular mechanism of metabolites of these specific species of *lactobacilli*. It should be noted that these two *lactobacilli* are not considered as the microbial flora of the oral cavity. In the present study, the cytotoxic effects of two *lactobacilli* which are present in the oral cavity (including saliva and the surface of the tongue) as well as in dental caries have been studied.

*L. fermentum* is one of the oral *lactobacilli* that is naturally present in the mouth. This *Lactobacillus* has shown high antioxidant properties among other species of human oral *lactobacilli* [23]. Studies in mice have shown the preventive effects of *L. fermentum* on colorectal cancer. This *Lactobacillus* in combination with some other *lactobacilli* has caused apoptosis increase and proliferation decrease in colorectal cancer cells. The significant preservation of normal colon cells is due to the toxic effects of these *lactobacilli* on cancer cells. Therefore, *L. fermentum* has been proposed as a type of biotherapy in colorectal cancers [24, 25]. Preservation of normal colon cells from the toxic effects of this *Lactobacillus* is a remarkable point. Therefore, *L. fermentum* has been proposed as a type of biotherapy in colorectal cancers [24, 25].

*L. fermentum* CQPC08 has been shown to increase concentrations of anticancer cytokines G-CSF and GM-CSF, thus blocking the reduction in immunity triggered by tongue cancer. This *lactobacilli* has also enhanced the activities of superoxide dismutase and glutathione peroxidase and diminished concentration of malondialdehyde in the tissue samples of the animal model of tongue cancer, thus defeating the oxidative stress injury in the tissues [26].

*L. crispatus* is another *Lactobacillus* with a possible role in the treatment of cancer. In the recently published articles, injecting this bacterium into the mice diagnosed with breast cancer has caused tumor size reduction and survival improvement. The results of the research on this *Lactobacillus* have proven the antiproliferative effect of this bacterium on the MDA-MB-231 cell line of breast cancer. This *Lactobacillus* has also caused a reduction in the expression of a group of cancer-related genes called cancer-testis antigens (CTA) in breast cancer cells [27]. Since expression of these genes is associated with high invasion of cancer cells and worse patient prognosis, *L. crispatus*-associated reduction in their expression caused can be a promising new method in the treatment of some cancers. In another study, mentioned bacterial supernatants reduced expression of HPV E6 oncogene in cervical cancer cells. This point is very important in its potential in the treatment of cancers caused by human papillomavirus in the mouth and cervix [28]. It has been shown that *L. crispatus* has cytotoxic effects on cervical cancer cells and reduces HPV virus oncogenes and cell cycle-related genes [29]. Since human papillomavirus is the etiologic cause of a significant percentage of cervical and oropharyngeal cancers [1], studies performed on the effect of *L. crispatus* on cervical cancers could pave the way for additional research studies on the control and treatment of oropharyngeal cancers.

The results of this study showed that *L. fermentum* and *L. crispatus* did not inhibit the progression of HN5 oral cancer cells. In other words, although with increasing concentration of *lactobacilli*, their toxic effects on cells increase, and these results are consistently less than the effects of MRS and MRSPH media. To prove the effects of *lactobacilli* on inhibiting the growth of cancer cells, the amount of growth inhibition in the presence of *lactobacilli* must be more than the culture media. The highest inhibition of cancer cell proliferation was obtained from the acidic culture medium. Taherian et al. showed that *lactobacilli* have different effects on different types of cancer cells. In other words, the decreased expression of genes associated with poor prognosis (such as S6K1) has been different in various categories of cancers [30]. This highlights the need to recognize and pay attention to the specific genetic characteristics of each tumor in adopting treatment methods.

Therefore, we suggest to assess the effect of these *lactobacilli* on different types of oral squamous cell carcinoma. The remarkable thing about the effect of *lactobacilli* is that their cytotoxic effects on various types of cancers are different. Another important point is that some oropharyngeal cancers are directly related to the human papillomavirus. These oral cancers have completely different clinical, histological, and prognostic features from other oral carcinomas [31]. Due to the specific effect of *L. crispatus* on reducing the incidence of HPV-related oncogenes, which has been proven in previous studies [29], a study aimed at investigating the effect of *L. crispatus* on HPV-induced oral cancer cell line is strongly recommended.

The next step after this study is to investigate the effects of other *lactobacilli* and oral cavity bacteria on the HN5 cell line to obtain an accurate list of *lactobacilli* or other bacteria that have a lethal effect on oral cancer cells or those effective on the progression of cancer. Since previous studies on the effects of *lactobacilli* on oral cancer have been limited to only two studies, several other studies are needed and recommended in this field.

In brief, we demonstrated no significant effect of two *lactobacilli* strains on an SCC cell line. Since the experimental design of this study has been simple, this study has not fully appraised the effect of *L. fermentum* and *L. crispatus* on this oral SCC line in vitro. Thus, we suggest further experiments to assess expression of possibly affected genes by these two strains of *lactobacilli*.

## Figures and Tables

**Table 1:** IC50 of cancer cells in different environments.

Concentration	MRS	MP	*L. crispatus*	*L. fermentum*

5	85	75	89	93
10	57	43	73	80
20	45	35	56	65
30	21	12	25	27
40	14	8	17	19
50	5	2	8	11

## Data Availability

The data used to support the findings of this study are available from Dr. Sepideh Mokhtari upon request.
